# Evaluation of trajectory analysis for disease risk assessment: a scoping review

**DOI:** 10.1093/jamia/ocaf208

**Published:** 2025-11-26

**Authors:** Freya Pollington, Spiros C Denaxas, Kezhi Li, Johan H Thygesen, Georgios Lyratzopoulos, Becky White

**Affiliations:** Epidemiology of Cancer Healthcare and Outcomes (ECHO) Research Group, Department of Behavioural Science and Health, Institute of Epidemiology & Health Care, University College London, London WC1E 7HB, United Kingdom; Institute of Health Informatics (IHI), University College London, London NW1 2DA, United Kingdom; BHF Data Science Centre, Health Data Research UK, London NW1 2BE, United Kingdom; Institute of Health Informatics (IHI), University College London, London NW1 2DA, United Kingdom; Institute of Health Informatics (IHI), University College London, London NW1 2DA, United Kingdom; Epidemiology of Cancer Healthcare and Outcomes (ECHO) Research Group, Department of Behavioural Science and Health, Institute of Epidemiology & Health Care, University College London, London WC1E 7HB, United Kingdom; Epidemiology of Cancer Healthcare and Outcomes (ECHO) Research Group, Department of Behavioural Science and Health, Institute of Epidemiology & Health Care, University College London, London WC1E 7HB, United Kingdom

**Keywords:** electronic health records, risk prediction, diagnosis codes, deep learning

## Abstract

**Objectives:**

Increasingly, structured longitudinal electronic health records (EHRs) are being harnessed to predict risk of having present but as yet undetected disease by analyzing “patient trajectories.” Trajectory studies explore clinical event associations, characterize disease trajectories, and enhance risk prediction. This scoping review assesses study characteristics and objectives, identifies model types, and appraises model performance and reporting.

**Materials and Methods:**

We conducted a scoping review, focused on a PubMed and Web of Science search for studies using temporal EHR sequences to identify disease signatures or predict disease presence.

**Results:**

We identified 62 studies. Statistical methods, such as testing temporal associations were primarily used for clustering, while deep learning models focused on outcome prediction. Sixty-five percent of studies used secondary care data, with the most common outcomes being disease agnostic (39%) and cardiovascular disease (20%). Forty-eight studies aimed at risk prediction, with 50% comparing trajectory-based models to static baselines. Among 31 studies reporting area under the curve (AUC), temporal models showed moderate performance gains (relative/absolute AUC: median 5.7%/4.2%, range −2.6% to 58.9%/−2.3% to 33.0%).

**Discussion:**

Trajectory studies are increasing in volume, but lacking in application to primary care datasets, a diverse set of diseases, external validation, and consideration of clinical applicability.

**Conclusion:**

While the field’s nascency hinders firm conclusions, there are promising results across a range of model types and objectives. Continued research from diverse perspectives will help determine whether this growing field can deliver meaningful clinical benefits.

## Introduction

A patient trajectory refers to a sequence of clinical events a patient experiences over time. The terms “disease trajectory” and “patient trajectory” have become increasingly present in the literature over the last decade, although alternative descriptors include “sequence,” “history,” or “longitudinal electronic health record (EHR).”^1–4^

**Figure 1.**
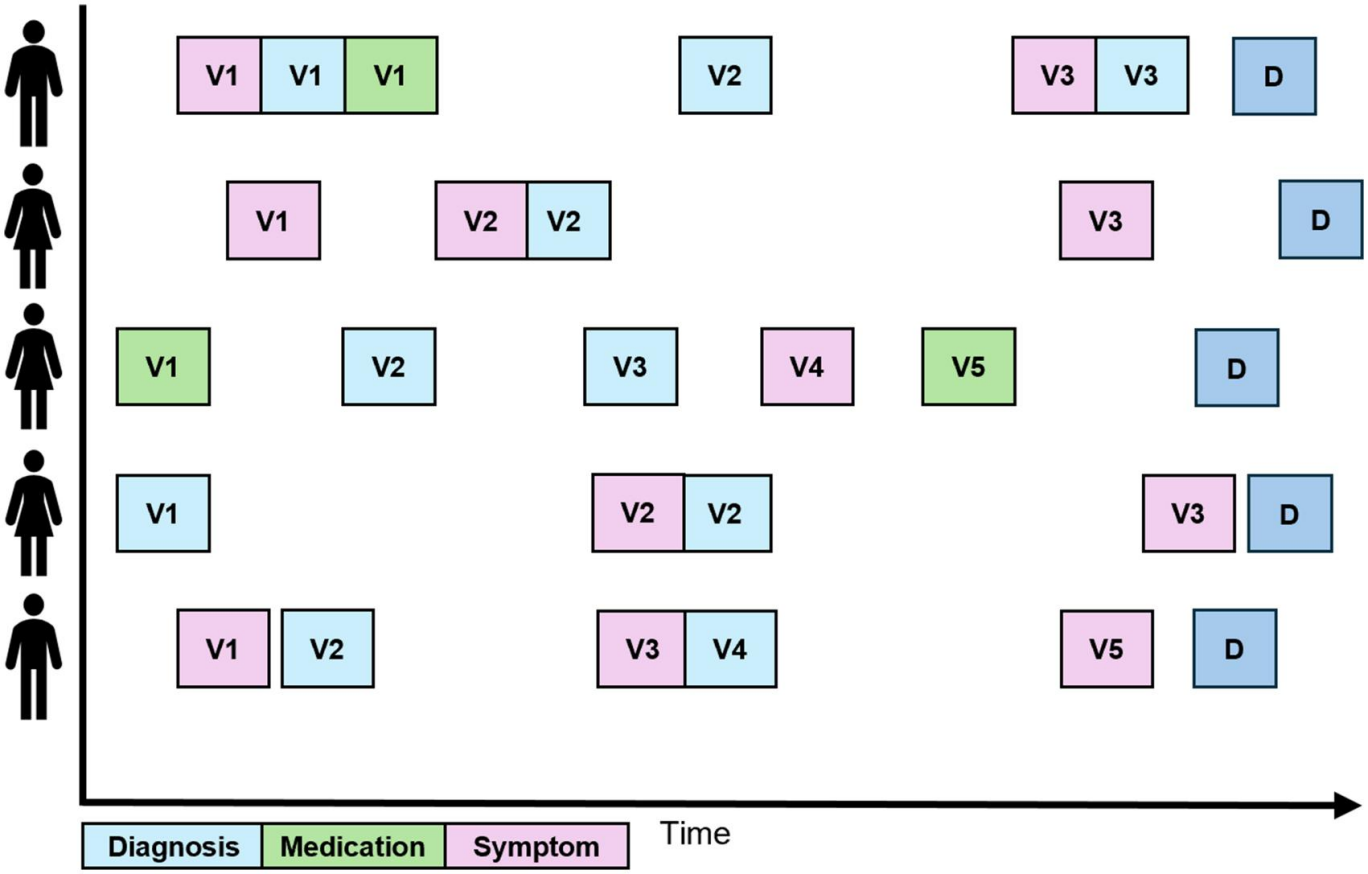
Graphic representation to exemplify patient trajectories for multiple individuals, which all end at the same diagnosis and have varying combinations of feature inputs and clinical visit timepoints (*V_x_*).

Figure 1 depicts the example of patient trajectories in the lead up to a common diagnosis “D.” The theory of trajectory research is that there could be shared information within the histories which is indicative of the final diagnosis, but on different time scales. Traditional (nonlongitudinal) risk prediction studies such as those by White et al^5^ and tools such as QRISK^6^ usually simplify these trajectories dramatically; for example, by identifying whether patients at time *t* had a symptom recorded within a specific period prior. In contrast, longitudinal methods offer greater potential to capture the specific timing and combination of predictors.

Various statistical methods have been applied to longitudinal data for disease prognostication, risk prediction, and readmission.^7–10^ These include frequentist and Bayesian approaches such as dynamic landmark models,^11^^,^^12^ and multistate models.^13^^,^^14^ However, these methods typically ignore temporal relationships, limiting model capability.

Machine learning (ML) techniques, including neural networks (NNs), random forests, and ensemble methods, offer new possibilities for temporal modeling in health care.^15^ Machine learning models handle high-dimensional datasets with irregularly recorded covariates, improving prediction accuracy without strong parametric assumptions. Deep learning (DL) has further revolutionized health-care analytics, enhancing diagnostic accuracy and personalized treatment.^16–18^ While its application to medical imaging, genomics, and unstructured data is relatively well established, comparatively less research focuses on structured EHR datasets.

Several reviews have examined longitudinal modeling of EHR data with varying perspectives. Many focus exclusively on DL methods,^4^^,^^19–23^ others include additional ML methods,^3^^,^^15^^,^^24^^,^^25^ and at least 1 excludes DL.^26^ A broad review encompassing statistical, ML and DL models is needed to compare performance and assess improvements over static models.

Additionally, previous reviews included free-text clinical data, but researcher access to free-text data can be severely limited in some health-care systems, for example in the United Kingdom and European Union, due to General Data Protection Regulation and other data protection legislation.^27–31^ In these health-care systems, population-level analysis must be conducted using structured data only. Therefore, a review was needed summarizing the temporal methods available to researchers who only have access to structured data.

Our aims included: identifying trajectory studies used in diagnostic research and their characteristics (data type, preprocessing techniques, objectives); providing an overview of methods to model trajectories; and appraising model performance, validation, and reporting where applicable.

## Methods

This scoping review followed the Preferred Reporting Items for Systematic Reviews and Meta-Analyses extension for scoping reviews (PRISMA-ScR), with a checklist shown in Table S3.^32^ A PubMed and Web of Science search was conducted for publications between January 1, 2014 and March 26, 2025, with inclusion terms falling into 3 categories: temporal/DL, medical records, and disease diagnosis. Disease diagnosis was favored over mortality and readmission given the methodological differences such as variables of interest and length of trajectory which can complicate their joint consideration. The full list was developed iteratively with exclusion terms added to increase relevance of papers to screen; both are detailed in Tables S1 and S2. Publications were then excluded based on the following criteria:

1. The study had an irrelevant objective or method
  - The study did not use methods which analyze patient sequences and retain temporality.
  - The study did not examine disease diagnosis, instead predicting clinical events, or disease progression.
2. The study did not use structured EHR data.
3. The study was not primary research.

**Figure 2.**
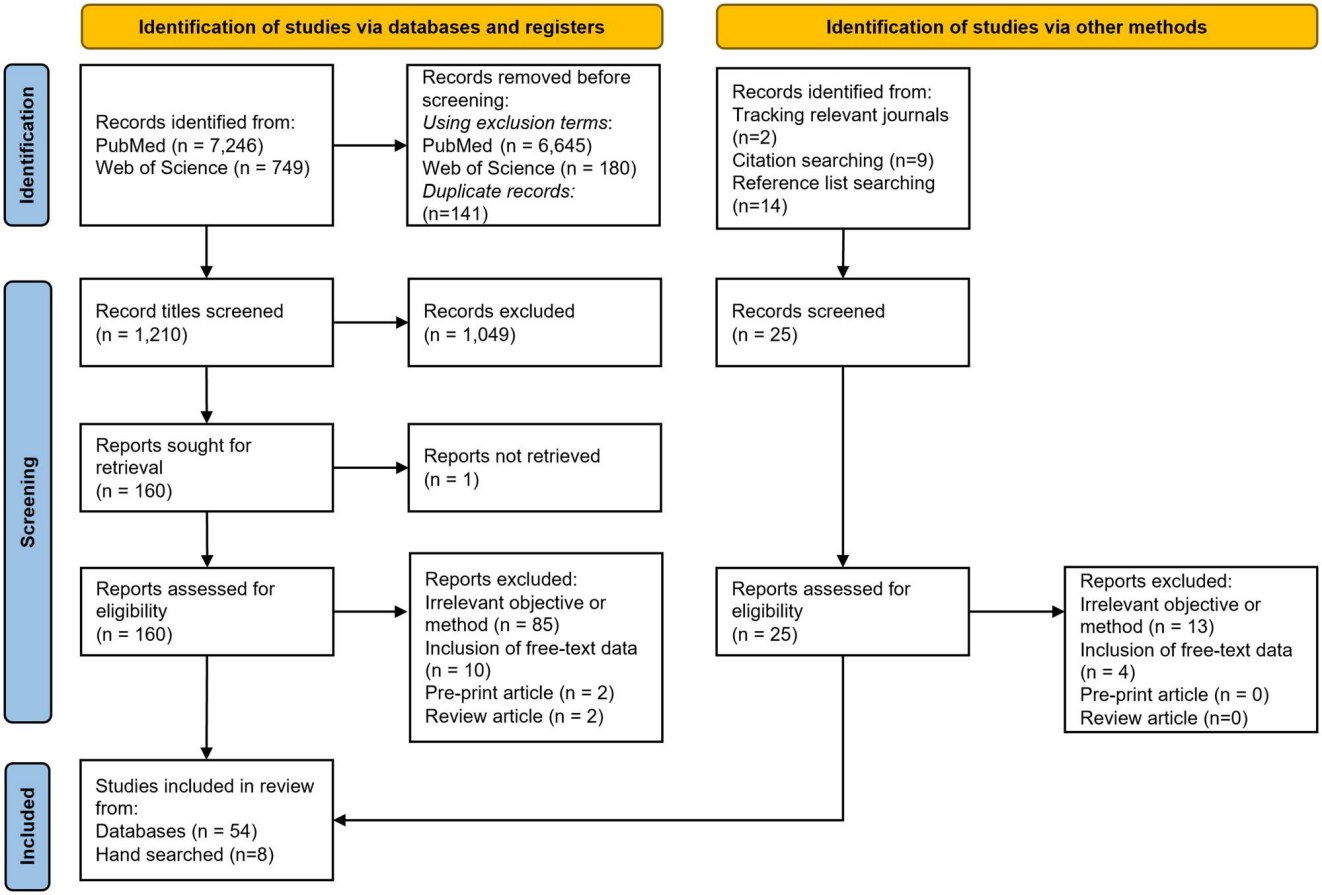
PRISMA flowchart displaying publication selection process.

After title, abstract, and full paper screening, 54 studies were retained. An additional 8 studies were included through tracking relevant journals, and citation and reference searching in papers of interest. This process is summarized in Figure 2. Table S4 summarizes the key elements of the included publications which are discussed in the following review sections.

## Trajectory model types

**Figure 3.**
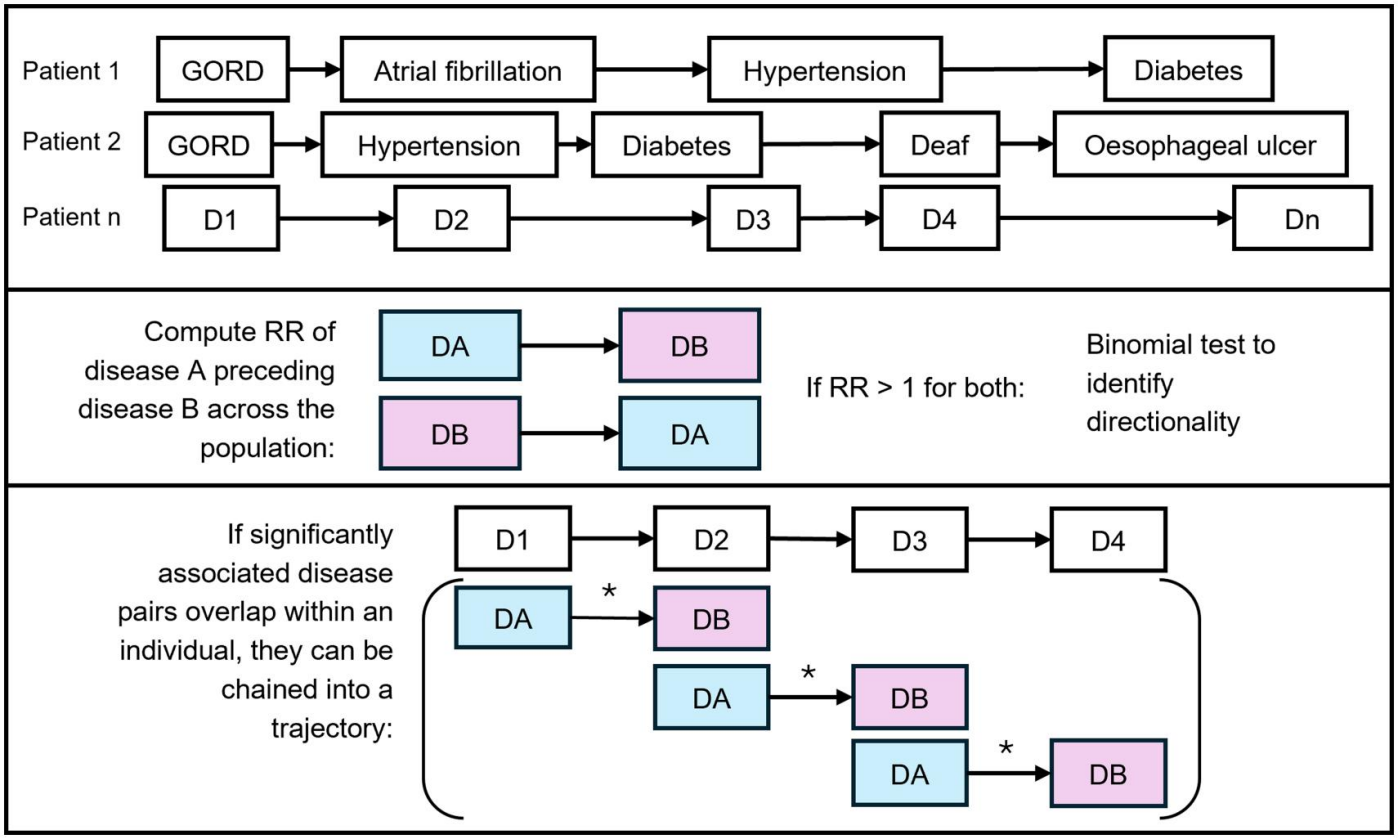
Graphic representation to display the process of evaluating disease trajectories using relative risk calculations. The top section demonstrates the example of disease records for individual patients across time on an unspecified axis. The middle section demonstrates how for each possible pair of diseases across the population, the relative risk (RR) of 1 disease occurring after another can be calculated (by comparison to an unexposed group, that is, the “risk” of disease A [DA] being followed by disease B [DB] against the “risk” of DA not being followed by DB). For disease pairs with RRs >1 (indicating a positive directional association), a binomial test is applied to identify directionality. The bottom section shows how a disease trajectory can be formed by chaining together pairs of directionally associated diseases for the pairs with a significant association that overlap, that is, in an example patient with D1–D4, if each pair in the sequence is found to be directionally associated at the population level, the pairs can be chained.

**Figure 4.**
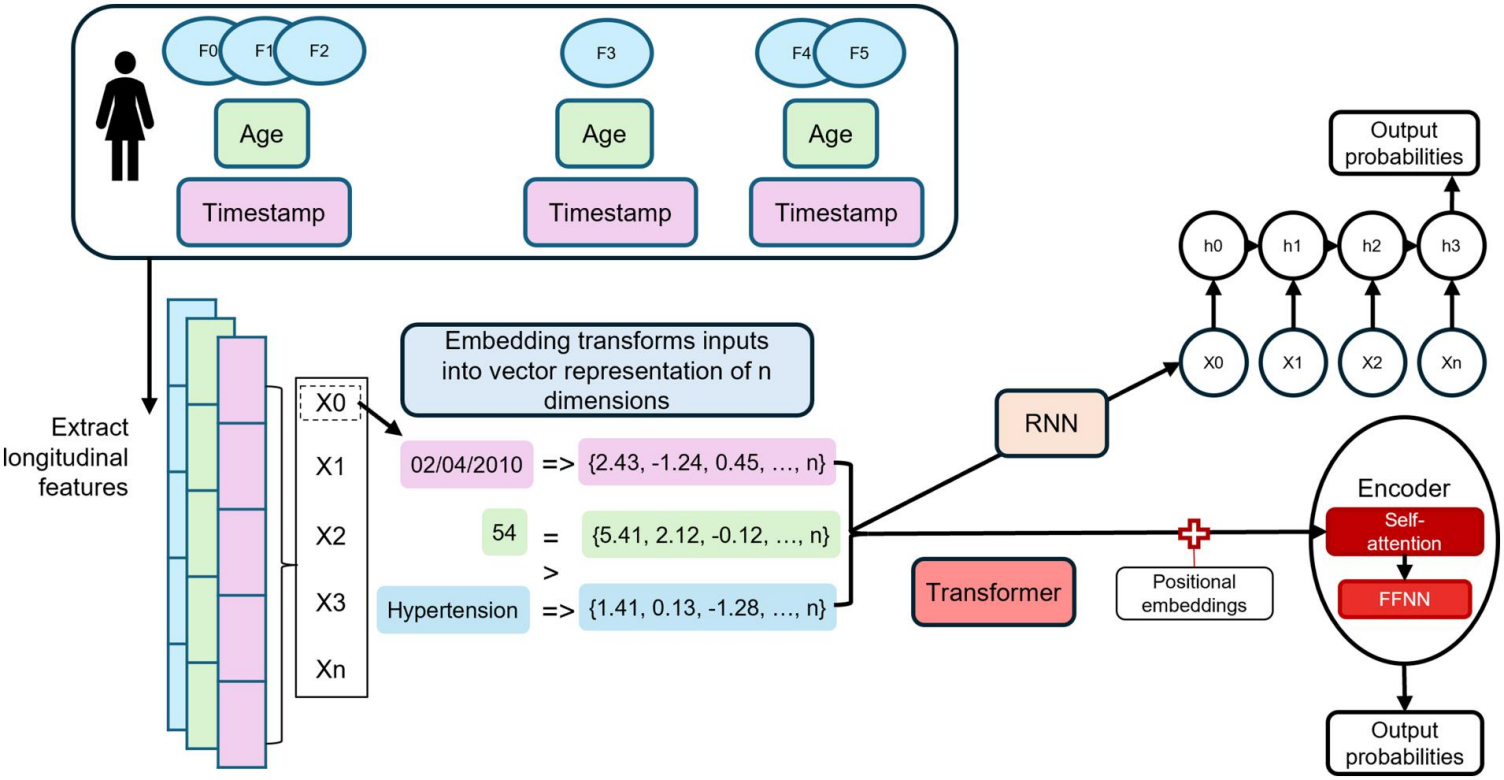
Graphic representation to display the process of evaluating disease trajectories using the 2 most common deep learning methods: recurrent neural networks and transformers. This simplified diagram demonstrates how a patient electronic health record made up of features, age, and a timestamp at each interaction with health-care can be extracted, preprocessed with embedding, and fed into neural network structures to predict an outcome of interest. Embedding layers are created for the sequence of features, age, and visit date in this example, meaning numeric vectors are created to capture semantic relationships. The RNN model takes this input and processes values in the sequence step by step, updating a hidden state (*h_x_*) at each step. The transformer model instead processes sequences in parallel, using self-attention to weight relationships between all inputs, and can include positional embeddings to allow the sequence order to be recognized.

This section will describe model types used in the reviewed studies, split into statistical and DL categories, to provide an understanding of these models prior to description of study and model characteristics in further sections. We have opted to present the statistical and DL methods under separate sections to reflect the lack of overlap in the existing evidence field. A basic depiction of the most common statistical and DL methods is shown in Figures 3 and 4.

### Statistical methods

Most studies in this review using statistical methods chain disease pairs, calculate risk ratios of the 2 occurring in sequence, and apply a statistical test to confirm directionality.

The first study to introduce “disease trajectory” analysis, was by Jensen et al using Danish EHRs covering 6 million patients.^33^ They linked disease pairs (disease A [DA], disease B [DB]) and calculated the relative risk (RR) of sequential diagnosis in both “directions,” then performed a binomial test to ascertain whether DA preceded DB significantly, or vice versa for those where directionality was unclear. Pairs were chained together into longer trajectories. The Markov Cluster Algorithm then identified disease clusters, and their distribution across trajectories. This resulted in trajectory clusters, identifying “central” disease(s) and related diseases.

This method, with modifications, has been adopted in subsequent studies.^34–42^ For instance, Paik et al and Taylor et al constructed directed acyclic graphs (DAGs), identified significant diseases and formed trajectories, the latter applied to excluding diagnoses distal to dementia.^37^^,^^40^ Giannoula et al followed similar steps, clustering using dynamic time warping, a technique which finds similarity between sequences with overlapping but unaligned features.^39^^,^^43^ This enabled identification of sex-specific directional comorbidities.

Oh et al combined sequence pattern mining with a likelihood function to estimate disease risk.^44^ They concatenate previous disease events with a newly developed disease, both at study baseline and in the follow-up window. They then chain relevant pairs together and test for the relationship between the pair, finally calculating the partial likelihood (the joint probability of outcome occurrence with stated baseline diseases and trajectories) to rank disease trajectories.

### DL methods

#### Recurrent neural network architectures

Recurrent neural networks (RNNs) process sequential data by retaining information across time steps through hidden states, enabling them to capture temporal dynamics.^45^ Unlike feedforward networks, RNNs have cycles that create a form of memory. However, they struggle with long sequences due to the vanishing/exploding gradient problem, leading to short-term memory.^45^ Long short-term memory (LSTM) models address this by introducing memory cells with gates that regulate information flow: forget (discard), input (store), and output (pass forward).^46^ To reduce computational demands, gated recurrent units (GRUs) simplify this design with only reset and update gates, while retaining comparable performance.^47^

Early DL applications to sequential EHR data often used RNNs. Choi et al developed DoctorAI, a GRU-based model predicting next disease or medication code, and time until the prediction.^1^ They later showed a performance improvement for GRU predicting initial heart failure diagnosis compared to static methods.^48^

Grout et al applied the Word2Vec algorithm and a bidirectional GRU model for prediction of type II diabetes, chronic obstructive pulmonary disorder, hypertension, and acute myocardial infarction.^49^^,^^50^ The bidirectional GRU processes input sequences in both forward and backward directions through separate layers,^51^ then combines representations at each time step for an aggregated output. To improve clinical interpretability, SHapley Additive exPlanations (SHAP) values quantified feature contributions to predictions.^52^

#### Convolutional neural network architectures

Convolutional neural networks (CNNs) most often applied to data such as medical images can also model temporal structure using 1D convolutional windows.^53^^,^^54^ These windows slide along the time axis to capture local patterns, with pooling layers reducing dimensionality. Extracted features are then used for prediction, and static variables (eg, demographics) can be added through connected layers. One publication utilized a CNN structure alone—Xception,^55^ aiming to predict lung cancer risk. Three publications combined a CNN with either a variation of LSTM^56^^,^^57^ or an attention mechanism.^58^

#### Architectures with attention

Recurrent neural networks tend to emphasize recent events, potentially undervaluing distant but informative ones. Attention mechanisms address this by assigning weights to inputs, capturing both short- and long-term dependencies.^45^^,^^59^ At each step, attention weights are calculated to determine the most relevant input elements for the current output. Transformer models extend this with self-attention, allowing each element to attend to all others and model dependencies regardless of distance.^60^

Early attention use was seen in DeepCare, an LSTM-based model with pooling to highlight critical diagnoses and interventions.^61^ This was a multioutcome model focusing on future diagnosis in diabetes and mental health patients. Choi et al predict heart failure with RETAIN, a reverse-time attention mechanism consisting of RNN and attention layers, prioritizing recent clinical visits.^62^

BEHRT is a transformer-based model inspired by Google’s bidirectional encoder representations from transformers (BERT), for natural language processing tasks.^63^^,^^64^ BEHRT was evaluated on Clinical Practice Research Datalink (CPRD) data, taking International Classification of Diseases 10 (ICD-10) and Read code representations, age, and sequence position as inputs to predict diseases in either 6 or 12 months, or at the next visit.

The most common outcomes for models with attention include heart failure/Cardiovascular diseases (CVD) and next clinical code. Three transformer-based models have been developed for pancreatic cancer prediction, and 2 for lung cancer, all improving predictions in comparison to baseline models.^2^^,^^65–68^

Beyond RNN subtypes, attention mechanisms have been integrated into other models that would not typically have that capability including general adversarial networks and DAGs.^69^^,^^70^

## Attributes of the included studies

The studies reviewed demonstrated wide variation in data sources, cohort characteristics, and study objectives. Below, we outline differences in data types and demographics used, the range of dataset sizes, and the diverse predictive aims across studies.

### Data characteristics

**Figure 5.**
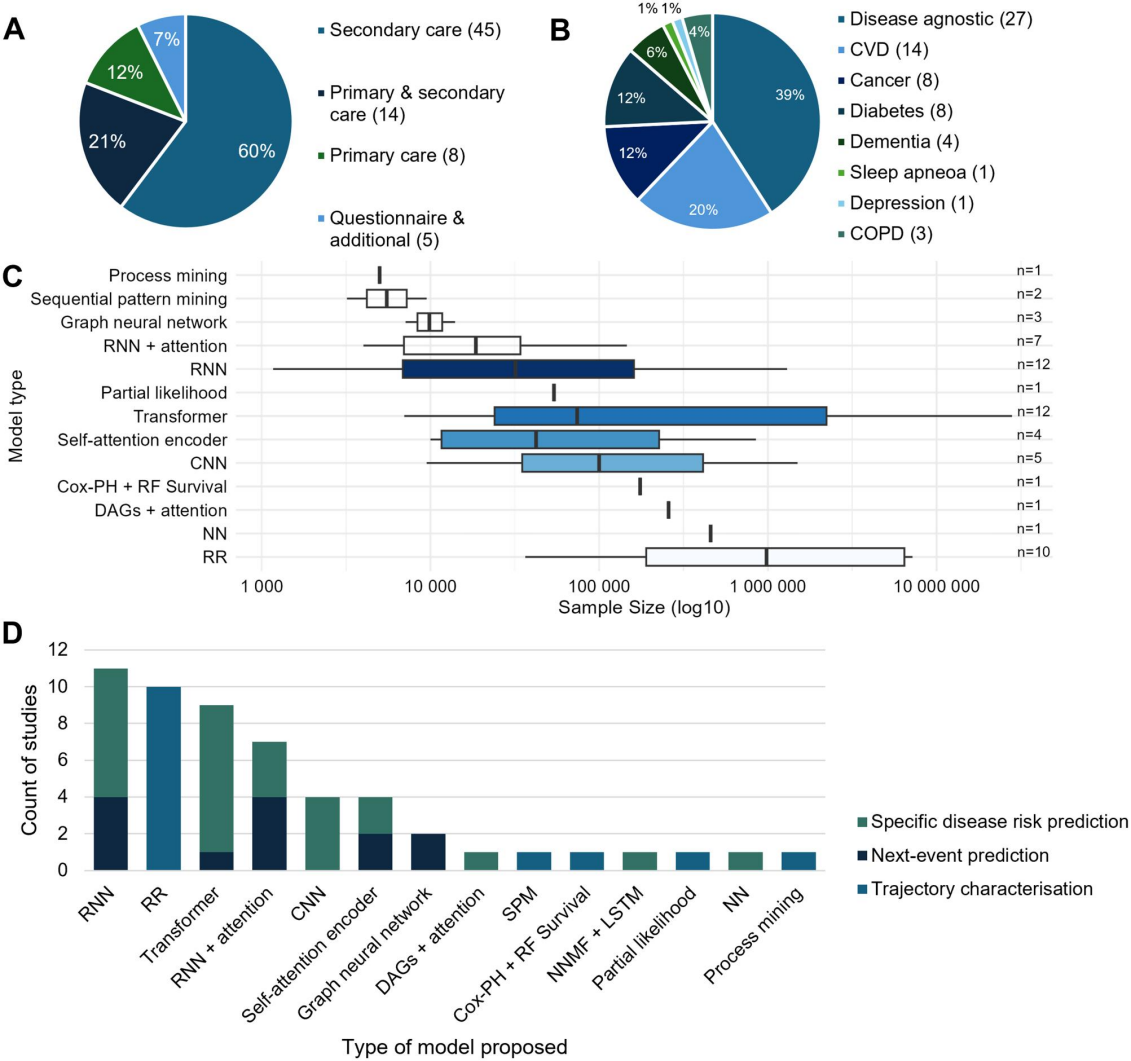
The proportion of data source types used across studies, if more than 1 data type was used, both are included in the count (A). The disease outcomes of trajectory model studies and their proportions, if more than 1 outcome was stated they all contribute to the count of the relevant outcomes (B). A boxplot to display the distribution of sample sizes for each of the model architecture types used across studies. If a study used multiple data sources, or individual sources for different disease outcomes, the largest sample size was displayed. The number to the right of each pair of bars is the number of studies for each model type (C). A stacked bar plot to show the spread of study objective categories (specific disease risk prediction, next-event prediction, trajectory characterization) by trajectory model type (D). Abbreviations: COPD, chronic obstructive pulmonary disease; CNN, convolutional neural network; DAG, directed acyclic graph; LSTM, long short-term memory; NN, neural network; NNMF, nonnegative matrix factorization; PH, proportional hazard; RF, random forest; RNN, recurrent neural network; RR, relative risk; SPM, sequence pattern mining.

The type and quality of EHR data is heterogeneous compared to other clinical data types like medical images. We classified studies as relating to either primary or secondary care, based on the data source used. This distinction is important in several countries where primary and secondary health care are delivered in separate settings; and therefore, studies of disease risk originating from these different settings can yield varied findings. Some studies were classified as both, if originating from medical centers that deliver both primary and secondary care services, or from studies that combine multiple data sources. Most studies included in this review used secondary care data (60%), primarily with ICD-10 codes. Data source proportions are displayed in Figure 5A. Other types of variation in data sources also existed; for instance, data could derive from billing within a national single-payer system or a multipayer (insurance-based) system.^71^

Demographics were inconsistently included. Age was an input in 49% of studies; however, some transformer-based models contextualized time differently or used specific age groups.^72^ Sex was included in 52% of studies, and ethnicity, race, and sociodemographic variables were rarely included.

Sample sizes varied greatly. Thirteen studies included <10 000 individuals,^48^^,^^61^^,^^62^^,^^66^^,^^73–81^ while 10 studies exceeded 1 million.^2^^,^^33–35^^,^^37^^,^^42^^,^^55^^,^^63^^,^^65^^,^^66^  Figure 5C displays average sample sizes split by model type. Transformer models had the largest range of sample size, skewed by a few large studies. The median showed RR-based models had larger samples, driven by the development of this technique by Danish researchers with access to the Danish National Patient Registry. No clear pattern emerged between sample size and model type if considering a split into “statistical’ and “DL.”

### Aims and outcomes of reviewed studies

Study objectives fell into 3 categories: identifying disease associations (trajectory characterization); predicting the next medical event; and forecasting specific disease risk. For the latter 2 categories, these can be further split into identifying present but as yet undiagnosed disease, and assessment of future disease risk. While all studies involved disease association testing or risk prediction, some did attempt additional prediction tasks for outcomes such as mortality.^82^

The spread of outcomes across all studies is shown in Figure 5B, with a large proportion being disease agnostic or focusing on CVD.

Some DL models aimed to predict the next diagnosis,^63^^,^^83–85^ while others focused on the disease classification. For instance, TransformEHR classified risk of pancreatic cancer and intentional self-harm in patients with posttraumatic stress disorder.^2^

Many studies calculating RRs had a disease agnostic outlook. For example, Paik et al aimed to identify disease trajectories which were associated with other diseases or death, searching for any unknown associations.^37^ While they discovered an association between schizophrenia and rhabdomyolysis which became their headline finding, they did not hypothesize this at baseline. In contrast, Beck et al applied the methodology specifically to diabetes and sleep apnea to identify if there is any directionality in the association previously found between the 2 diseases.^34^ Similarly, the Patient Trajectory Analysis Library (PTRA) model developed by Herzeel et al was designed to be applicable to a range of diseases, but the authors tested it on the ability to extract medically relevant trajectories for bladder cancer.^38^ The distribution of overall study objective (outlined above) is shown by trajectory model type in Figure 5D.

## Methods for simplification of the medical coding system

Structured EHR data typically consists of time-stamped medical codes for each patient ID. Common coding systems in the reviewed studies include the ICD-9 or -10,^86^ Read codes,^87^ SNOMED-CT,^88^ Current Procedural Terminology,^89^ and Generic Product Identifier.^90^ These systems contain thousands of hierarchical codes which can complicate model building and interpretation.

To address this, authors applied methods to simplify or create representations of these codes. One method was using Clinical Classification Software which reduces sparsity in ICD-10 codes by encoding them into fewer, higher order, categories.^91^ An encoding processes the input sequence to capture contextual dependencies between the input tokens. Five studies use this method.^38^^,^^48^^,^^62^^,^^70^^,^^75^ Another common technique was truncating ICD-10 codes to 2-4 digits.^2^^,^^33–37^^,^^39–41^^,^^56^^,^^61^^,^^65^^,^^74^^,^^76^^,^^85^^,^^92–94^ One study mapped Read and ICD-10 codes to CALIBER codes, a University College London open-access dictionary that condensed 10k+ diagnosis codes into 301 phenotypes.^63^^,^^95^ The simplification methods for each study are indicated in Table S4 where present.

Transformers use encoding layers to transform the token embeddings and learn contextual relationships via self-attention. Token embeddings, applied in all deep-learning models, transform discrete input tokens into continuous vector representations.^45^ Embeddings can be trained with the model, or use pretrained embeddings, for example, Word2Vec^49^ and GloVe (global vectors for word representation),^96^ which predict context words for a given target word or words. Studies in this review either used embeddings alone, or combined them with sparsity reduction. For instance, Choi et al applied the skip-gram pretrained embedding,^1^^,^^48^ whereas Li et al,^63^ Yang et al,^2^ Placido et al,^65^ and Pham et al^61^ trained their own embedding layers.

## Temporal representation of medical codes and patients

Including the time dimension increases modeling complexity in comparison to static representations of EHRs. Given this challenge, varying methods have been applied and will be summarized below.

### Directional flow of information and time windows

Nondeep learning models typically identify directionality between diagnosis codes within a certain time frame. This approach often fails to account for the exact time between events and usually only considers the first instance of a code, excluding repeats in the sequence.

Jensen et al applied a 5-year window to identify diagnosis pairs with RRs >1 and chained without further temporal consideration, only allowing first disease occurrence for each patient.^33^ Beck et al followed this methodology,^34^ as did Singhal et al, with additional analysis of time from comorbidity to diagnosis.^36^ Paik et al introduced a filter to only include diagnoses within 1 year of each other.^37^

### Including time-related features in the model

Some models included input-specific temporal information, particularly in transformer architectures, as RNNs process input data sequentially, updating the hidden state at each time step. Each hidden state acts as an encoding, representing the current token in the context of all previous tokens. For example, DoctorAI included time-stamped ICD-9, medication and procedure codes in a GRU structure.^1^ Later testing showed minimal performance improvement when including the time between events and the index date.^48^ DeepCare also incorporated time-stamped events with a pooling technique.^61^ Its LSTM model used diagnosis codes for each time step and elapsed time between admissions, with look-back periods of 12, 24 months and the full history for prognosis prediction.

In transformer models, positional embeddings are commonly added to the token embeddings to account for token order, as transformers process tokens in parallel.^60^ These embeddings are learned representations of position.

BEHRT modeled temporal data using age and visit order,^63^ embedding disease codes, relative position, age, and visit segment. Rao et al applied BEHRT to heart failure prediction, finding the addition of calendar year improved performance over age alone.^97^ Other studies implemented time-related embeddings using visit date,^2^ order and position of codes within a visit,^66^ and age at and the time difference between each diagnosis.^65^

## Performance and sensitivity analyses

The following section covers the 48 studies with predictive outcomes. Table 1 summarizes performance metrics used, displaying best prediction window results where applicable. Definitions for each metric are stated in Table S5.

**Table 1.** Performance of predictive models included in the present review.

Paper	Model	Static contrast	Trajectory contrast	AUC/AUROC	Precision	Recall	F1 score	RMSE	AU-PR	Accuracy	Recall @30	Accuracy @30
Choi et al^1^ (skip-gram)	RNN-2 hidden layer	Y	Y	—	—	—	—	—	—	—	0.796	—
Choi et al^62^	RNN + attention	Y	Y	0.87	—	—	—	—	—	—	—	—
Choi et al^48^	GRU	Y	Y	0.88	—	—	—	—	—	—	—	—
Pham et al^61^	DeepCare (LSTM + attention)	N	Y	—	66.2	52.7	—	—	—	—	—	—
Choi et al^70^ (heart failure)	GRAM	N	Y	0.845	—	—	—	—	—	—	—	—
Suo et al^98^	CNN + time fusion	N	N	—	—	—	—	—	—	0.774	—	—
Song *et al*^99^	Transformer	Y	N	0.771	—	—	—	—	—	—	—	—
Teoh^76^	GRU + FCNN	N	Y	0.669	—	—	—	—	—	—	—	—
Tang et al^56^ (CKD, |AKD)	CNN-LSTM	Y	N	0.872, 0.863	—	—	—	—	0.172, 0.488	—	—	—
Suo et al^100^	CNN	Y	N	—	0.852	0.841	0.844	—	—	0.844	—	—
Rasmy et al^101^	RETAIN (RNN)	N	Y	0.822	—	—	—	—	—	—	—	—
Li et al^102^	SPM	N	Y	0.86	—	—	—	—	—	—	—	—
Chen et al^75^	GRU	Y	N	0.791	—	—	—	—	—	—	—	—
Li et al^63^	BEHRT (transformer)	N	Y	0.9	—	—	—	—	—	—	—	—
Wang et al^103^	Wide and deep (LSTM) + NNMF	Y	Y	—	—	—	—	—	0.308	—	—	—
Wang et al^74^	LSTM, 4-digit ICD, MIMIC	N	Y	—	0.997	0.993	0.99	—	—	—	—	—
Wang et al^58^	FCNBLA	N	Y	—	0.923	0.905	0.913	—	—	0.928	—	—
Zeng et al^104^ (MIMIC-III, |PFK)	MSAM	Y	Y	—	—	—	—	—	—	—	0.683, 0.795	—
Li et al^94^	Graph CNN	N	Y	—	—	—	—	—	—	—	—	0.818
Ye et al^104^ (HF, |KD, dementia)	LSAN	Y	Y	0.846, 0.867, 0.831	0.621, 0.651, 0.584	0.626, 0.672, 0.616	0.623, 0.661, 0.599					
Estiri et al^78^ (CHF, COPD, RA, TID, TIID, UC)	tSPM	N	Y	0.826, 0.788, 0.793, 0.904, 0.808, 0.849	—	—	—	—	—	—	—	—
Boursalie et al^106^ (ICD-10 chapter 1)	DT-THRE (transformer)	N	Y	—	0.927	0.738	—	—	—	—	—	—
Poulain et al^73^	BEHRT (transformer)	N	Y	—	—	—	—	0.145	—	—	—	—
Meng et al^93^	HCET + attention	Y	Y	0.81	—	—	—	—	0.73	—	—	—
Yeh et al^55^	Xception (CNN)	Y	N	0.902	—	—	—	—	—	—	—	—
An et al^92^	LSTM + attention	Y	Y	0.79	0.616	0.707	0.659	—	—	—	—	—
Rasmy et al^66^ (diabetic heart failure, pancreatic cancer)	Med-BERT (transformer)	Y	Y	0.852, 0.817	—	—	—	—	—	—	—	—
Kwak et al^107^	SAF-RNN	Y	Y	0.839	—	—	—	—	0.661	—	—	—
An et al^57^	TAMDUR	N	Y	0.937	0.878	0.884	0.881	—	—	—	—	—
Poulain et al^69^	CEHR-GAN-BERT	Y	Y	0.877	—	—	0.574	—	0.646	—	—	—
Park et al^108^	NN + composite embeddings	N	N	0.858	—	—	—	—	0.435	—	—	—
Liu et al^82^	CATNet (transformer)	N	Y	0.952	—	—	—	—	0.425	—	—	—
Javidi et al^72^ (ages 3-12, 5-12, 3-8)	Time-series forest-CNN	N	N	0.72, 0.71, 0.69	—	—	—	—	—	—	—	—
Wu et al^109^ (coronary heart disease, hypertension)	LSTM + attention	Y	Y	0.925, 0.858	—	—	—	—	0.798, 0.820	—	—	—
Rao et al^97^	BEHRT (transformer)	N	Y	0.93	—	—	—	—	0.69	—	—	—
Liu et al^110^ (MIMIC-III, eICU)	MCF-LSTM-multibelt fusion	N	Y	0.950, 0.966	—	—	—	—	0.400, 0.593	—	—	—
Sun et al^80^	Graph neural network	N	Y	—	—	—	0.213	—	—	—	0.355	0.471
Placido et al^65^	CancerRiskNet (transformer)	Y	Y	0.934	0.186	0.165	—	—	—	—	—	—
Al Olaimat et al^111^	PPAD	Y	Y	—	—	∼0.960	—	—	—	—	—	—
Yang et al^2^ (pancreatic cancer)	TransformEHR (transformer)	Y	Y	0.82	—	—	—	—	0.786	—	—	—
Chen et al^67^	ViT-transformer	Y	Y	0.668	—	—	—	—	—	—	—	—
Wang et al^68^ (not lung cancer)	MedAlbert + LR	Y	N	0.924	0.999	0.834	0.909	—	—	—	—	—
Grout et al^50^ (diabetes)	Bi-GRU	N	N	0.917	—	—	—	—	—	—	—	—
Al Olaimat et al^112^	TA-RNN	Y	Y	—	—	∼0.96	—	—	—	—	—	—
Wang et al^113^ (MIMIC-III, MedClin)	Mdpg	N	Y	—	—	—	—	—	—	—	—	0.878, 0.958
Wang et al^114^	MB-TCN-TC	N	N	—	0.723	0.734	0.728	—	—	—	—	—
Luo et al^79^	Graph transformer	N	Y	—	—	—	0.878	—	—	—	—	—
Wang et al^81^	DKGC-LSTM	Y	Y	0.89	—	0.79	—	—	—	0.77	—	—

The proposed headline model has its performance reported. Where multiple outcomes/datasets were reported, the category of reported performance in this table is indicated in brackets alongside paper details. Certain metrics were not included in the table including negative log likelihood,^62^ average precision score,^63^ varying values of *k* for Recall@*k*,^104^ and F2.^112^

Abbreviations: AKD, acute kidney disease; BERT, bidirectional encoder representations from transformers; CKD, chronic kidney disease; FCNN, fully connected neural network; GRU, gated recurrent unit; LSTM, long short-term memory; N, no; NNMF, non-negative matrix factorization; PPAD, predicting progression of Alzheimer’s disease; RNN, recurrent neural network; SAF-RNN, self-attention fusion RNN; SPM, sequence pattern mining; TA-RNN, time-aware RNN; TAMDUR, Time-Aware Multitype Data fUsion Representation learning framework; TID, type 1 diabetes; TIID, type 2 diabetes; ViT, vision transformer; Y, yes.

The area under the curve (AUC)/area under the receiver operating characteristic curve (AUROC) was the most reported metric (63% of risk prediction studies). The use of other performance metrics was sporadic. Twenty-eight percent of studies reported only 1 performance metric.

**Figure 6.**
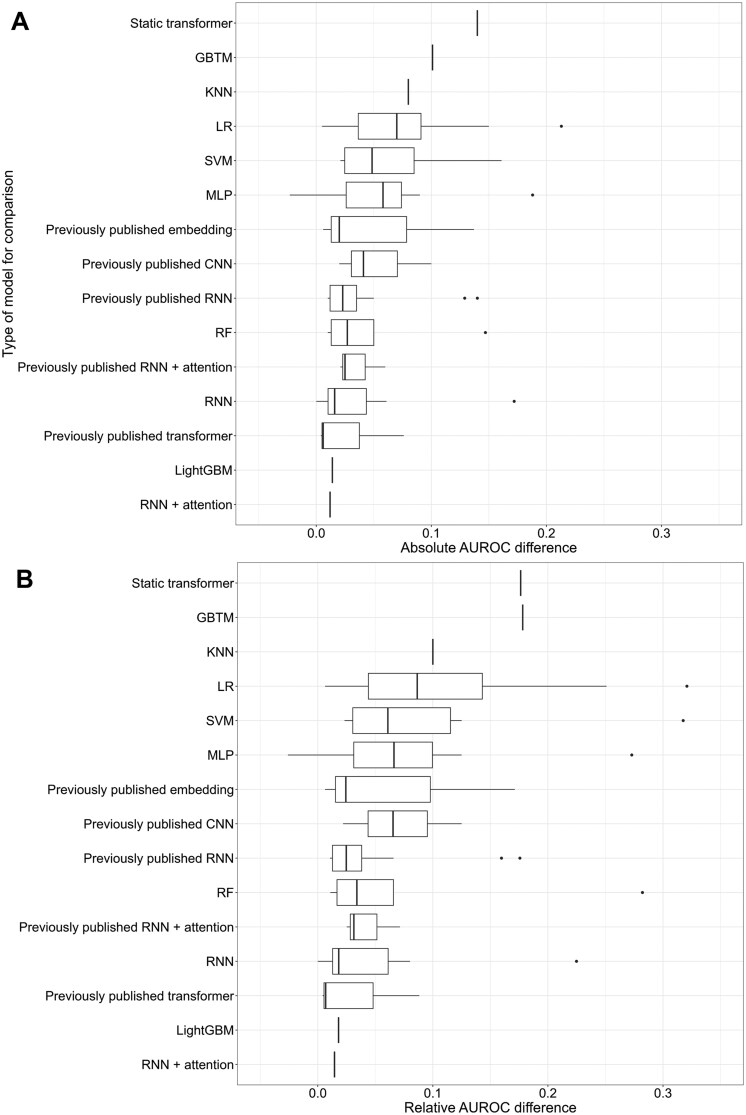
Boxplots displaying the distribution of the absolute (A) and relative (B) difference in AUC performance for trajectory-based models in comparison to the author’s chosen baselines, for studies reporting AUC (*n* = 31). Baseline model types are shown on the *Y*-axis. Abbreviations: AUROC, area under the receiver operating characteristic curve; GBTM, group-based trajectory model; KNN, *K*-nearest neighbor; LightGBM, light gradient boosted machine; LR, linear regression; MLP, multilayer perceptron; RF, random forest; RNN, recurrent neural network.

The best-performing model achieved AUC: 0.966,^110^ while the worst-performing model achieved only 0.668.^67^ Generally, proposed models outperformed preexisting trajectory-based or static models, with greater improvements over unoptimized baselines. Figure 6 displays the distribution of relative and absolute AUC differences for trajectory models compared to various baselines. Twenty-four studies (50%) evaluated static models on their dataset and prediction outcome, and 37 evaluated baseline temporal models (77%). Twenty-four studies evaluated previously published trajectory models on their dataset and prediction outcome, sometimes alongside static or unoptimized baselines.^2^^,^^57^^,^^63^^,^^66^^,^^67^^,^^69^^,^^72^^,^^73^^,^^79–82^^,^^92^^,^^93^^,^^97^^,^^104–107^^,^^110^^,^^112^^,^^113^^,^^115^^,^^116^ Some studies found minimal improvements,^56^^,^^69^^,^^82^^,^^92^^,^^104^^,^^112^ while others showed marked gains, such as TransformEHR, which improved pancreatic cancer classification by 3.5%, 6.4%, and 11.4% over BERT, LSTM, and linear regression (LR), respectively.^2^ Tang et al found that artificial intelligence (AI) models incorporating temporal information did not outperform a static multilayer perceptron (MLP) model across multiple diseases.^56^

Model performance also depended on observation period length, and preprocessing methods. For example, Choi et al compared GRU with LR, MLP, Support vector machine (SVM), and *K*-nearest neighbor (KNN).^48^ For an 18-month observation window, GRU performed 5.9% better than MLP. Static models aggregated the data, representing code frequency over the period. All models were found to perform best with 12 months of observation data and a 3-month prediction window. Placido et al found that removing codes within 3 months prediagnosis reduced performance.^65^ Similar finding was found by Kwak et al who removed 7- and 14-days of data before CVD diagnosis.^107^ Meng et al tested sliding 6-month windows, finding performance increased with proximity of observation period and diagnosis.^93^

Chen et al assessed whether clinical event frequency in a trajectory varied for cases vs controls, and if this impacted the prediction algorithm.^75^ They found optimal performance for 5-10 encounters over 2 years. When training set sizes fell below ∼25 000, GRU underperformed compared to RF and LR models, dependent on the input features. They also examined observation and prediction window lengths, noting that longer windows, especially for prediction, improved GRU performance relative to RF and LR.

In terms of validation of the proposed models, 17 (35%) studies performed external validation, the rest either performed internal validation or did not validate results at all (for nonrisk prediction). The importance of model validation will be expanded on in the “Discussion” section.

## Discussion

This review identified a range of trajectory-based methods applied to EHR data, their objectives and associated challenges of capturing temporal patterns. Both DL and non-DL methods have advanced, with a notable increase in frequency of DL application. Consistent with previous reviews, while certain DL models show notable performance gains, this is not found in all cases.

Studies came from diverse health-care settings and data sources. Some studies had large sample sizes, while others lacked sufficient data to model rare codes and capture true longitudinal dependencies. Sex was included less frequently than expected given its dominant role as an epidemiological effect modifier.^117–119^ In addition, the lack of inclusion of ethnicity, race, and sociodemographics may limit model generalizability and potentially exacerbate systemic biases, raising concerns about fairness in model predictions across diverse populations.^120^

Electronic Health Record sequence length and preprocessing techniques also varied. Statistical models generally performed better with shorter observation windows, whereas DL models, particularly those using self-attention, captured long-term dependencies more effectively and improved risk prediction. Performance declined when excluding data immediately prior to diagnosis.^65^^,^^93^^,^^107^ Placido et al noted that disease codes right before diagnosis may clearly signal disease, prompting clinican referral without the need for inferential analysis.

Establishing whether trajectory analysis offers a tangible benefit over static models, or those that do not fully capture the longitudinal dependencies in the data, is crucial. Therefore, studies should compare the performance of a new trajectory model to both existing static forms as well as competing longitudinal forms. This may identify computational differences, variation in identified variables of interest, data requirements and risk prediction performance. Further research in this field should also report on as wide a range of metrics as possible so that easier identification of performance could be ascertained. Presenting 1 metric can mislead interpretation and lead to evaluation bias particularly in unbalanced datasets such as in the case of rare diseases.^3^^,^^4^^,^^19^^,^^24^^,^^121^ Studies reporting high accuracy and moderate AUC despite very low true positive rates and F1 scores demonstrate that AUC can mask poor performance when events are rare.^122^

External validation was lacking, increasing uncertainty in the applicability of models to different data settings. Accessibility and budgeting for a secondary data source can be challenging. Despite this, it is crucial for identifying model generalizability to different populations.^123^

External validation could additionally be enabled by authors sharing open-source code, allowing for reviews that compare primary studies. A review by Solares et al externally validated 4 DL models which used EHR data and had been published by 2016: They used CPRD, and open-source code where available or the model parameters stated in the chosen papers.^19^ Further research of this kind which allows true comparisons to be drawn between proposed models is needed. A modern approach to this is federated learning, which offers a way to train models across diverse datasets while preserving privacy.^124^ Building global risk models for common outcomes of interest could improve performance and generalizability. It may also help to mitigate risk of spurious disease associations that arise from biases in EHR coding. These can vary according to the health-care system (eg, single payer vs insurance-based) and reflect organizational or insurance policies.^71^^,^^125^

Alternative approaches to model validation may entail manual review of patient records by trained staff or cross-linking to additional data such as genetic biobanks (only feasible within certain cohort studies).^125^

No studies used decision-analytic or clinical utility assessment. Measuring net benefit or cost-effectiveness by incorporating predicted risks with clinical or economic outcomes may improve implementation.^126^ TRIPOD+AI encourages transparent reporting and outlines the need for discussing “Usability of the model in the context of current care.”^127^ Guidelines such as these should be followed to allow authors to demonstrate potential usefulness.^128^ Diseases lacking obvious early signs (eg, pancreatic cancer, Alzheimer’s) may particularly benefit from trajectory analysis by improving patient stratification,^129^ but implementation in practice requires collaboration with clinical stakeholders.

The clinical applications of the longitudinal approaches included in this review can be grouped into 3 categories:

1. **Identifying trajectories for specific disease(s), for example,** Herzeel et al^38^—Inform diagnostic guidelines, such as the National Institute of Health and Care Excellence guidelines for suspected cancer referral, as these are organized by disease.
2. **Predicting the risk of a specific disease in a specified time frame, given a patient’s clinical trajectory, for example,** Placido et al^65^—Inform clinician referral decisions for presenting patients, using all information from individuals’ medical records to generate risk estimates, and could be used to further enhance existing, but static clinical decision-making tools, such as QCancer.
3. **Predicting the most likely disease in a specified time frame, given a patient’s clinical trajectory, for example,** Wang et al^74^—Inform clinicians about which diseases to investigate first when a patient presents. However, how to explicitly translate these methods into clinical practice is so far unclear in the literature.

It appears that there is more than sufficient scope to produce novel literature, either via primary research studies, application of preexisting methods to a new problem, or validation of a range of models on 1 dataset to compare performance as seen in Solares et al^19^ Research on primary care datasets is lacking, as has been picked up by previous review articles. In addition, while CVDs are well represented, other diseases have not yet been thoroughly examined.

This review has limitations. The search was limited to PubMed and Web of Science, potentially omitting studies in alternative repositories. Only studies using structured data were included, excluding those using free-text or multimodal data to reflect the needs of researchers with limited free-text access. Future reviews could explore models tailored to free-text sources, which often requires natural language processing preprocessing. Lastly, varied performance metrics and baseline models hindered detailed comparisons, so AUC/AUROC was chosen as a primary metric for simpler cross-study comparisons.

In summary, disease trajectory analysis is an emerging and promising field, with interest partly fueled by the ongoing excitement around AI. Continued research from diverse perspectives will help determine whether this growing field can deliver meaningful clinical benefits.

## Acknowledgments

The primary author would like to thank the funders Health Data Research UK and the co-authors for their support in the production of this manuscript.

## Supplementary Material

ocaf208_Supplementary_Data

## Data Availability

No new data were generated or analyzed in support of this research.
