# Supplementary material for: Evaluation of trajectory analysis for disease risk assessment: a scoping review
Source: J Am Med Inform Assoc. 2025 Nov 26;33(2):521–35. doi: 10.1093/jamia/ocaf208 (PMC12844584; doi:10.1093/jamia/ocaf208)
Supplement: ocaf208_Supplementary_Data [file ocaf208_supplementary_data.docx]

# **SUPPLEMENTARY MATERIAL**

**Supplementary table 1:** Inclusion terms used in a PubMed and Web of Science search.

| **Inclusion terms** |
| --- |
| ("temporal" OR "trajector*" OR "deep" OR "dynamic" OR "transformer") |
| ("electronic health record*" OR "EHRs" OR "EHR" OR "registry" OR "electronic medical record*" OR "EMR" OR "EMRs" OR "health dataset") |
| ("diseas*" OR "cancer" OR "diagnos*") |

**Supplementary table 2:** Exclusion terms used in a PubMed and Web of Science search, shown in the PubMed format.

| **Exclusion terms** | | |
| --- | --- | --- |
| GWAS | gene* | polygen* |
| proteom* | DNA | RNA |
| disability | metabolom* | autopsy |
| prevalence [Title] | hospital [Title] | readmission [Title] |
| pathogenesis | incidence [Title] | uptake |
| prognostic | screening programme | forecasting |
| trend* | multimodal | life course |
| mendelian | geograph* | psychological |
| preoperative | trial | operation* |
| injur* | transplant* | traum* |
| pathology | drug* | chemo* |
| radio* | treatment [Title] | serum |
| visual | imag* | photo* |
| biomarker* | clinical notes | clinical text |
| infect* | in vivo | cytokines |
| pregnan* | vaccin* | inflamm* |
| Systematic Review [Publication Type] | Review [Publication Type] |  |

**Supplementary table 3:** Preferred Reporting Items for Systematic review and Meta-analyses extension for Scoping Reviews (PRISMA-ScR) Checklist.


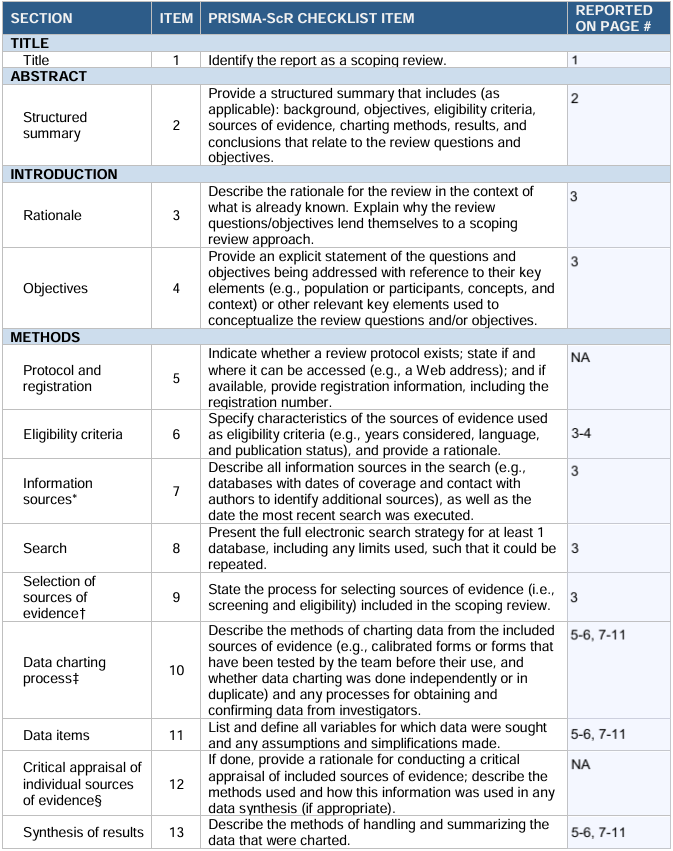


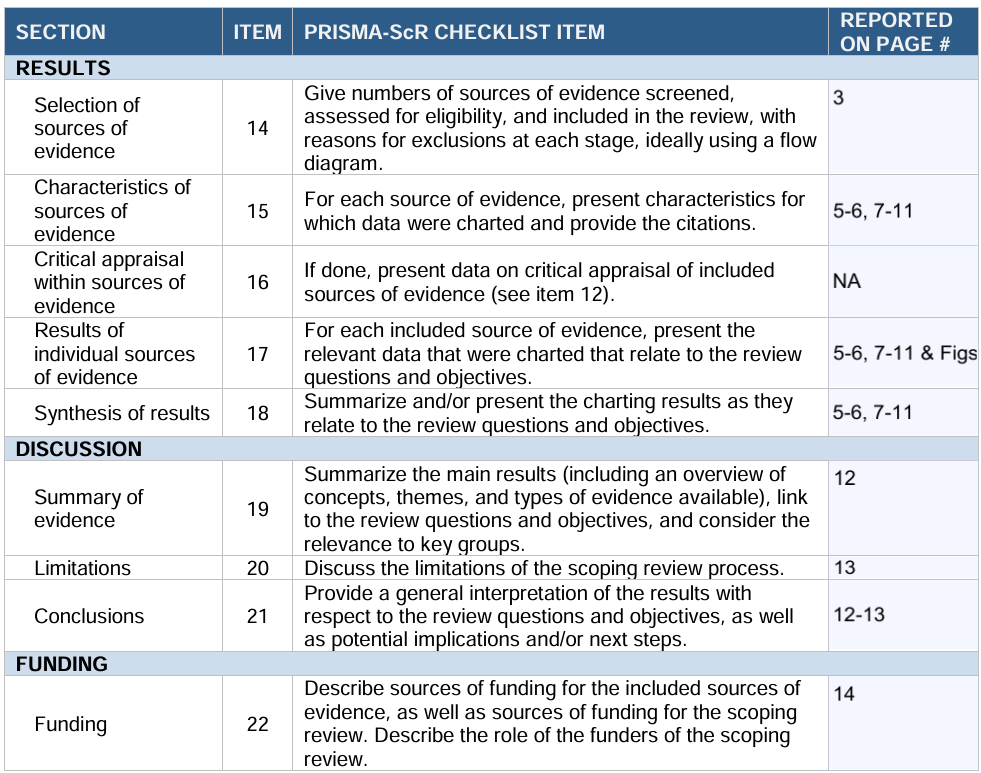


**Supplementary table 4.** Characteristics of the publications included in this review. The columns ‘Training/ (validation/) test split’ and ‘Comparison with other model types?’ are relevant only to models with prediction outcomes.

| **Year** | **First Author** | **Paper title** | **Model type** | **Dataset** | **Sample size** | **Observation window** | **Predictors/ stratifiers** | **Outcome** | **Training/ (validation/) test split** | **Comparison with other model types?** | **External validation?** |
| --- | --- | --- | --- | --- | --- | --- | --- | --- | --- | --- | --- |
| 2014 | Jensen.A | Temporal disease trajectories condensed from population-wide registry data covering 6.2 million patients (33) | RR | DNPR (secondary care) | 6 million | 5 years | ICD-10 codes (3 digits), sex, age | Multi/ none | NA | N | Y |
| 2016 | Choi.E | Doctor AI: Predicting Clinical Events via Recurrent Neural Networks (1) | GRU | Sutter Health Palo Alto Medical Foundation (primary care) | 263,706 (avg. 54.6 visits per person) | - | Time-stamped ICD-9, GPI medication codes, CPT procedure codes – grouped into a 1778-dimensional vector OR skip-gram | Diagnoses/ medications for next visit | 85/15 | MLP, LR | N |
| 2017 | Choi.E | Using recurrent neural network models for early detection of heart failure onset (48) | GRU | Sutter Health Palo Alto Medical Foundation (primary care) | 4178 case, 29139 control, 265336 for training medical concept vectors | 12-18 months | Time-stamped ICD-9 CCS, GPI medication codes, CPT procedure codes – one-hot-encoded OR skip-gram | Heart failure | 5/1/1 | LR, MLP, SVM, KNN | N |
| 2017 | Pham.T | Predicting healthcare trajectories from medical records: A deep learning approach (61) | LSTM + attention | Australian hospital (secondary care) | 7191 (diabetes), 6109 (mental health) | 12 years | ICD-10, procedure and medication codes (2 digits) | Next diagnosis | 4/1/1 | Markov, RF, SVM. RNN | N |
| 2017 | Choi.E | RETAIN: An interpretable predictive model for healthcare using reverse time attention mechanism (62) | RNN + attention | Sutter Health Palo Alto Medical Foundation (primary care) | 3884 cases, 28903 controls | 18 months | ICD-9 grouped to CCS, GPI grouped to GPIDG, CPT grouped to CCS for CPT | Heart failure | 0.75/0.1/0.15 | LR, MLP, GRU, RNN + attention (one-layer single direction), RNN + attention (reverse-order) | N |
| 2017 | Beck.M | Temporal order of disease pairs affects subsequent disease trajectories: the case of diabetes and sleep apnoea (34) | RR | DNPR (secondary care) | 6.9 million | 5 years | ICD-10 (3 digits) | Diabetes, sleep apnoea | NA | NA | N |
| 2017 | Choi.E | GRAM: Graph-based attention model for healthcare representation learning (70) | DAGs + attention | Sutter Palo Alto Medical Foundation, MIMIC III (primary care, secondary care) | 258,000, 7,500 | 2+ codes | ICD-9 grouped into CCS | Heart failure | 75/10/15 | RNN variations | N |
| 2017 | Suo.QL | Personalized Disease Prediction Using a CNN-Based Similarity Learning Method (111) | CNN + time fusion | Unspecified HER (primary care, secondary care) | 100,000 | 2 years | ICD-9 (symptoms, diseases) | COPD, diabetes, obesity | 80/20 | N | N |
| 2018 | Song.HA | Attend and Diagnose: Clinical Time Series Analysis Using Attention Models (112) | Transformer | MIMIC-III (secondary care) | 33,798 | 2 weeks | ICD-9 | Multi | 60/13/27 | LR, LSTM | N |
| 2018 | Giannoula.A | Identifying temporal patterns in patient disease trajectories using dynamic time warping: a population-based study (39) | RR + Dynamic time warping | Catalan-wide clinical registry (CMBD) (secondary care) | 643,358 | 7 years | ICD-9 (3 digits), sex | Multi (comorbidity pairs or clusters of common disease trajectories) | NA | NA | N |
| 2018 | Teoh.D | Towards stroke prediction using electronic health records (80) | GRU, FCNN | Tsuyama Jifukai Tsuyama Chuo Hospital (secondary care) | 2725 cases, 5450 controls | 35 years for diseases, 20 years for exam results | ICD-10 (4 digits), laboratory results (both averaged at each age), sex, age, days since last diagnosis | Stroke | 70/15/15 | NA | N |
| 2018 | Tang.F | Predictive modelling in urgent care: a comparative study of machine learning approaches (56) | Bi-LSTM, CNN-LSTM | MIMIC-III (secondary care) | 30414 | >24 hrs | ICD-9 (3 digits), 6 vital signs, 13 laboratory tests, age, marital status, ethnicity, insurance information | One of 25 most commonly appearing conditions | ? | MLP | N |
| 2018 | Suo.Q | Deep Patient similarity learning for personalized healthcare (113) | CNN | Unspecified EHR (secondary care) | 9528 | 12 months | ICD-9 | Diabetes, obesity, COPD | 75/10/15 | ED, CS, LMNNC, ITML, GMML, SCML | N |
| 2018 | Rasmy.L | A study of generalizability of recurrent neural network-based predictive models for heart failure onset risk using a large and heterogeneous EHR data set (114) | RETAIN (GRU) | Cerner Health Facts (secondary care) | 152k cases, 1.2 million controls | 12 months | ICD-9/10 (CCS), medications, surgical procedures, laboratory tests | Heart failure | 70/10/20 | LR | Y (original dataset) |
| 2019 | Paik.H | Tracing diagnosis trajectories over millions of patients reveal an unexpected risk in schizophrenia (37) | RA + DAGs | Healthcare cost & Utilisation Project California SID (secondary care) | 1.5 mil | 1980-2010 (median 40.58 months) | ICD-9 (3 digits) | Multi (disease trajectories associated with other diseases or death) | NA | NA | Y |
| 2019 | Lademann.M | Incorporating symptom data in longitudinal disease trajectories for more detailed patient stratification (35) | RR | DNPR (secondary care) | 6.6 million | 5 years | ICD-10 (3 digits), sex, age | Multi | NA | NA | NA |
| 2019 | Li.J | Efficient Mining Template of Predictive Temporal Clinical Event Patterns from Patient Electronic Medical Records (115) | Sequential pattern mining + LR | Unspecified EHR data warehouse (secondary care) | 3196, 50 | 4 years | ICD-9 (grouped into eight disease categories), medications, lab tests, procedures, sex, date of birth, event type | Congestive heart failure | NA | NA | N |
| 2019 | Chen.R | Recurrent neural networks for early detection of heart failure from longitudinal electronic health record data: implications for temporal modelling with respect to time before diagnosis, data density, data quantity, and data type (79) | GRU | Sutter Palo Alto Medical Foundation (primary care) | 4370 cases, 30132 controls | 2 years | ICD-9 (CCS), medications (ATC), smoking, alcohol use, sexual history, demographics, vitals | Heart failure | 90/10 | RF, L1 Regularised regression | N |
| 2019 | Lin.H | Disease correlation network: a computational package for identifying temporal correlations between disease states from large-scale longitudinal medical records (130) | Cox-PH regression + RF survival analysis | Loyola University Chicago Medical Center (secondary care) | 175k | 10 years | Disease codes, date of first diagnosis, age, sex, race/ethnicity | Multi | NA | NA | N |
| 2020 | Li.Y | BEHRT: Transformer for Electronic Health Records (63) | Transformer | CPRD + HES (primary & secondary care) | 1.6 mil | >6 months or >12 months of data | ICD-10 (4 digits) and Read codes mapped to Calibre codes, age, encoding for position and visit segment combined into one embedding | Concepts in next visit, disease in 6 months, disease in 12 months | 80/10/10 | DeepR (131), RETAIN (62) | N |
| 2020 | Wang.Y | Enhancing model interpretability and accuracy for disease progression prediction via phenotype-based patient similarity learning (116) | Non-negative matrix factorisation + LSTM | IQVIA (primary care) | ? | 1 year | Diagnoses, prescriptions, procedures all aggregated to higher level categories, sex, age | Chronic lymphocytic leukaemia | 0.8/0.1/0.1 | CNN, RF, LR | N |
| 2020 | Wang.T | Long short-term memory recurrent neural networks for multiple diseases risk prediction by leveraging longitudinal medical records (78) | LSTM | MIMIC-III, GenCare (Shenzen China) (secondary care) | 7105, 4170 | 2001-2012, ? | ICD-9,10 (4 & 3 digits & top categories), age, sex, ethnicity, marital status, length of hospital stay | Multi (diagnoses in next visit) | 90/10 | GRU, RNN | Y |
| 2020 | Siggaard.T | Disease trajectory browser for exploring temporal, population-wide disease progression patterns in 7.2 million Danish patients (42) | RR | DNPR (secondary care) | 7.2 million | 24 years | ICD-10, sex | NA | NA | NA | NA |
| 2020 | Wang.T | Assistant diagnosis with Chinese electronic medical records based on CNN and BiLSTM with phrase-level and word level attentions (58) | CNN + BiLSTM + attention (FCNBLA) | Huangshi Central Hospital (secondary care) | 18601 | ? | Initial diagnosis, chief complaint, vital signs, specialist condition, general condition, lifestyle-related variations, medical history-related variables nutritional status, family history | 10 common diseases | 70/10/20 | SVM-TFIDF, CNN, RCNN, BiLSTM | N |
| 2020 | Zeng.X | Multilevel self-attention model and its use on medical risk prediction (104) | Multilevel self-attention model | MIMIC-III, Partner for Kids (primary & secondary care) | 7537, 146k | 1 year | ICD-9 (CCS), medications, procedures, age, sex | CCS diagnosis code groups in next visit | ? | TIMELINE (132), Dipole (133), Bi-RNN, RNN, MLP | Partner for Kids |
| 2020 | Jørgensen.I.F | Age-stratified longitudinal study of Alzheimer’s and vascular dementia patients (41) | RR | DNPR (secondary care) | 49112 Alzheimer’s, 24101 vascular dementia | 3, 5, or 10 years | ICD-10 (3 digits), age | Alzheimer’s disease, vascular dementia | NA | NA | N |
| 2020 | Kusuma.G | Process Mining of Disease Trajectories: A Feasibility Study (81) | Process mining | Simulated EHR dataset (secondary care) | 50 | ? | ICD-10 (3 digits), time of diagnosis | Multi | NA | NA | N |
| 2020 | Li.Y | Knowledge guided diagnosis prediction via graph spatial-temporal network (109) | Graph convolutional neural networks | MIMIC-III, Unspecified HER (secondary care) | 7499, 14060 | ? | ICD-9 (2 digits) | Multi | 75/10/15 | Dipole (133), GRAM (70), KAME (134) | N |
| 2020 | Ye.M | LSAN: Modeling Long-term Dependencies and Short-term Correlations with Hierarchical Attention for Risk Prediction (108) | Transformer | IQVIA (primary care) | 12,320 (HF), 11,240 (KD), 9,540 (Dementia) | ? | ICD-9 (symptoms, diseases) | Heart failure, Kidney disease, Dementia | ? | TIMELINE (132), T-LSTM (135), RetainVis (136), RETAIN (62), Dipole (133), GRU, LSTM, RF, LR, SVM | N |
| 2020 | Estiri.H | Transitive Sequential Pattern Mining for Discrete Clinical Data (82) | Transitive sequential pattern mining | Mass General Brigham Biobank | 6857 (CHF), 5107 (COPD), 4015 (RA), 3107 (Type I diabetes), 9500 (Type II diabetes), 1560 (Ulcerative colitis) | ? | ICD-9 (diagnoses, medications) | Congestive heart failure, COPD, Rheumatoid arthritis, Type I diabetes, Type II diabetes, Ulcerative colitis | NA | SPM | N |
| 2021 | Boursalie O | Decoder Transformer for Temporally-Embedded Health Outcome Predictions (110) | DT-THRE (transformer) | Unspecified EHR (secondary care) | 66906 | 2006-2017, 4+ visits | ICD-10 (22 chapter codes), imaging records (background effective dose), age, sex, time since last, visit number | ICD-10 chapter | 60/20/20 | Med-BERT(66) | N |
| 2021 | Poulain.R | Transformer-based multi-target regression on electronic health records for primordial prevention of cardiovascular disease (77) | BEHRT | All of Us Research Program (questionnaire + molecular + primary & secondary care) | 6993 | 3+ years | Diseases, prescriptions, (both grouped using ‘IsA’) relationship, lab tests, age, sex, race, ethnicity, position | 11 modifiable risk factors for CVD | 80/20 | RETAIN (62), Dipole (133), StageNet (137) | N |
| 2021 | Meng.Y | HCET: hierarchical clinical embedding with topic modelling on electronic health record for predicting depression (97) | Hierarchical clinical embedding (GRU-based) | Unspecified EHR | 10148 | 2006-2013: 2 weeks, 3 months, 6 months, 12 months windows pre-diagnosis/ EHR end | ICD-9 (3 digits), CPT, medications, sex, age, clinical notes | Depression | 70/10/20 | LASSO regression, SVM, MLP, RF, VAE + RF, MiME (138) | N |
| 2021 | Yeh.M.C | Artificial intelligence-based prediction of lung cancer risk using nonimaging electronic medical records: deep learning approach (55) | Xception (CNN) | Taiwan National Health Insurance Research Database (primary & secondary care) | 1,617 cases, 1423154 controls | 1999-2013: 3 years | ICD-9 (grouped into 1099 categories), medications (grouped into 830 categories), age, sex | Lung cancer | Pre-2012,post-2012 | NA | N |
| 2021 | An.Y | High-risk prediction of cardiovascular diseases via attention-based deep neural networks (96) | LSTM + attention | Xiangya medical dataset (secondary care) | 146k | >5 visits, prediction within 1 year | ICD-10 (3 digits), lab tests and demographics including age, sex, patient type, hospital visit times and surgery history | CVDs | 70/10/20 | Deepr (131), Dipole (133), R-MeHPAN (139), LR, SVM, RF, LightGMB | N |
| 2021 | Oh.W | A computational method for learning disease trajectories from partially observable EHR data (44) | Partial likelihood | Mayo clinic (primary & secondary care) | 53509 | 2005-2007 and 2012-2014 | Demographics (age, sex, BMI), diagnosis codes, vital signs, lab results, prescriptions | All diseases | NA | Causal graph learned by max-min hill climbing, dynamic bayes network, DBN + generating synthetic data | Y |
| 2021 | Rasmy.L | Med-BERT: pretrained contextualised embeddings on large-scale structured electronic health records for disease prediction (66) | Transformer | Cerner Health Facts (secondary care) | 28 million | 2000-2017 | Demographics (age, sex, race), ICD-9,10 codes | Diabetes heart failure, Pancreatic cancer | 70/10/20 | GRU, Bi-GRU, RETAIN (62), L2-regularised LR, RF | N |
|  |  |  |  | (primary & secondary care) |  |  |  |  |  |  |  |
| 2021 | Kwak.H | Interpretable disease prediction using heterogeneous patient records with self-attentive fusion encoder (105) | Self-attention fusion encoder + GRU | National Health Insurance Service South Korea (secondary care) | 798k cases, 68k controls | 2 years | Prescriptions, diagnoses, demographics (age, sex, residential area, income level) | CVD | 80/10/10 | Regularised LR, MLP, GRU, Patient2Vec, attentional RNN, GRU + bilinear self-attention | N |
| 2021 | An.Y | Time-Aware Multi-Type Data Fusion Representation Learning Framework for Risk Prediction of Cardiovascular Diseases (57) | CNN + Bi-LSTM | Xiangya Medical Dataset (secondary care) | 18k cases, 90k controls | >5 visits | Diagnosis codes, medication codes, laboratory tests, examination codes, sex, race, ethnicity, age | CVD | 80/10/10 | Dipole (133), DeepRisk (96), AdaCare (140), Timeline (132) | N |
| 2022 | Poulain.R | Few-shot learning with semi-supervised transformers for electronic health records (69) | General Adversarial Network + transformer | All of Us Research Program (questionnaire + primary & secondary care) | 29736 | <=3 years | Diseases, medications, procedures, sex, race, ethnicity, position | Heart failure | 70/10/20 | CEHR-BERT (141), BEHRT (63), Dipole (133), LR, MLP | N |
| 2022 | Park.J | Structured deep embedding model to generate composite clinical indices from electronic health records for early detection of pancreatic cancer (117) | NN | Columbia University Irving Medical Centre-New York Presbytarian Hospital (secondary care) | 458k | 2004-2021 | Laboratory measurements, controls adjusted for age, sex, ethnicity, | Pancreatic cancer | 80/10 | N | N |
| 2022 | Liu.S | CATNet: Cross-event attention-based time-aware network for medical event prediction (86) | Cross-event attention + GRU or LSTM or transformer | MIMIC-III, eICU Collaborative Research Dataset (secondary care) | 5438, 9215 | 2001-2012, 2014-2015 (=>2 visits) | ICD-9/10, medications, laboratory tests, procedures, mortality status | Medication, diagnosis, procedures, laboratory test | 80/10/10 | DoctorAI (1), T-LSTM (135), RGNN-TG-ATT (142), LSTM-DE (143), RetainVis (136), StageNet (137),ConCare (144), HiTANET (145) | Y |
| 2022 | Javidi.H | Identification of robust deep neural network models of longitudinal clinical measurements (72) | Time-series forest-convolutional neural networks | Cleveland Clinic baseline for simulated data (secondary care) | 35k | 1987-2020 | ICD-9/10, medications, BMI, demographics, laboratory results, age | Type 2 diabetes | 66/14/30 | Time-series forest-MLP, transformer, RNN-FCN, MLP, FCNN, ResNet (146), Gramian angular field CNN, convolutional-RNN (for simulation tasks not diabetes prediction) | N |
| 2022 | Wu.C | A method for the early prediction of chronic diseases based on short sequential medical data (118) | LSTM + attention | The First Affiliated Hospital, Zhejiang University School of Medicine (secondary care) | 35.6k | 0-1799 day range | Laboratory tests, ECG features, clinical observations, physical examinations, demographic features, age, sex | Diabetes, hypertension, coronary heart disease, arrhythmia, heat disease | 80/10/10 | RF, SVM, LSTM, LSTM-multi label, | N |
| 2022 | Rao.S | An Explainable Transformer-Based Deep Learning Model for the Prediction of Incident Heart Failure (101) | BEHRT (transformer) (63) | CPRD + HES (primary & secondary care) | 100k | >= 3 years | Diagnoses (Read codes, ICD-10), medications, age, calendar year | Heart failure | 60/20/20 | Deepr (131), RetainVis (136) | N |
| 2022 | Liu.S | Multi-channel fusion LSTM for medical event prediction using EHRs (102) | Multi-channel fusion (MCF) LSTM | MIMIC-III, eICU (secondary care) | 5438, 9215 | >=2 visits | ICD-9/10, medications, procedures, laboratory tests, sex, age, ethnicity, region | Next medical code | 80/10/10 | DoctorAI (1), RetainVis (136), HiTANet (145), T-LSTM (145), StageNet (137) | N |
| 2022 | Sun.Z | EHR2HG: Modeling of EHRs Data Based on Hypergraphs for Disease Prediction (84) | Hypergraph neural network | MIMIC-III (secondary care) | 7125 | ? | ICD-9 diagnoses | Multi | 84.25/1.75/14 | RETAIN(62), Deepr(131), GRAM(70), Dipole(133), Timeline(132), MedGCN(147), CGL(148) | N |
| 2023 | Placido.D | A deep learning algorithm to predict risk of pancreatic cancer from disease trajectories (65) | GRU or Transformer with FFN | DNPR (secondary care) | 6 million | 1977-2018 | ICD-8,9,10 (3 digits), age, time between diagnoses | Pancreatic cancer within incremental time windows | 80/10/10 | Bag-of-words, MLP | Y |
| 2023 | Al Olaimat.M | PPAD: a deep learning architecture to predict progression of Alzheimer’s disease (119) | RNN + MLP / RNN + AE | ADNI, National Alzheimer’s Coordinating Center (questionnaire & secondary care) | 1169 | ? | Dementia-related features, age, sex, education, ethnicity, race | AD | 70/30 | T-LSTM, RF, SVM | Y |
| 2023 | Herzeel.C | A software package for efficient patient trajectory analysis applied to analysing bladder cancer development (38) | RR + clustering | TriNetX Dataworks Network (primary & secondary care) | 128k | -2021 | ICD-10 (CCS), sex, age, BC treatment | Clusters of clinical findings | NA | - | N |
| 2023 | Singhal.P | DETECT: Feature extraction method for disease trajectory modelling in electronic health records (36) | RR | Penn Medicine Health System (secondary care) | 146k | 1998-2022 | ICD-10 (4 digits, must include hypertension code) | Multi (separate model for each) | NA | - | Y (simulated) |
| 2023 | Taylor.R.A | Dementia risk analysis using temporal event modelling on a large real-world dataset (40) | RR + dynamic time warping | Northeastern US emergency department (secondary care) | 420k | 2013-2022 | Epic procedural codes, medications, ICD-10 (3 digits) and lab, age, race and sex | Dementia | NA | NA | N |
| 2023 | Yang.Z | TransformEHR: transformer-based encoder-decoder generative model to enhance prediction of disease outcomes using electronic health record (2) | Transformer | VHA Corporate data warehouse (primary & secondary care) | 6.8 million | 2016-2019 (one outcome and one prior visit per patient) | Time-stamped ICD-10 (3 digits), days from last visit, sex, age, race, marital status | Multi (pancreatic cancer, intentional self-harm) | 70/10/20 | LR, LSTM, BERT without pre-training, pre-trained BERT (64) | Y |
| 2023 | Chen.H.Y | Lung cancer prediction using electronic claims records: a transformer-based approach (67) | Transformer | National Health Insurance Research Database (primary & secondary care) | 1949 cases, 80143 controls | 3 years | ICD-10 (chapters and lower level – 283 groups), ATC codes for medications (chapters, broader groups), sliding window of code occurrence, age, sex | Lung cancer | 60/20/20 | ViT-Transformer, CNN-LSTM, Xception (149), gradient-boosting decision tree, regularised regression, RF | Y (random sample of same population) |
| 2024 | Wang.L | Transformer-based deep learning model for the diagnosis of suspected lung cancer in primary care based on electronic health record data (68) | Transformer | Whole Systems Integrated Care Northwest London (primary care + secondary care) |  | 3 years within 1981-2020 | Read codes grouped to a higher level of hierarchy (symptoms, diagnoses, medications, procedures, sites of encounter, medical tests), age, sex, ethnicity, lifestyle factors, comorbidities | Lung cancer | 70/30 | LR | N |
|  |  |  |  |  | 89 cases, 7240 controls: 2932 with chronic respiratory conditions, 2030 with other cancers, 2279 with other conditions |  |  |  |  |  |  |
| 2024 | Grout.R | Predicting disease onset from electronic health records for population health management: a scalable and explainable Deep Learning approach (50) | Bi-GRU | Accenture AHA (secondary care) | 104k diabetes, 77k COPD, 289k hypertension, 76k myocardial infarction | 9 years observation, 3 years prediction | SNOMED codes for diagnosis and procedures, National Drug Code Directory Codes, Logical Observation Identifiers Names and Codes (observations), age, smoking status, alcohol consumption, race, sex, insurance type | Diabetes, COPD, hypertension, myocardial infarction | 90/5/5 | - | N |
| 2024 | Al Olaimat.M | TA-RNN: an attention-based time-aware recurrent neural network architecture for electronic health records (103) | RNN + attention | ADNI, MIMIC-III (questionnaire + molecular + secondary care) | 1169 ADNI \| 1205 ADNI, 6118 NACC, 7537 MIMIC-III | ? | Cognitive performance, MRI, sex, education years, ethnicity, race, time between visits | Clinical outcome at next visit (conversion of mild cognitive impairment to Alzheimer’s disease) | 70/30 ADNI \| all ADNI, all NACC \| 70/10/20 MIMIC-III | RF, SVM, Time aware-LSTM, PPAD (119) | Y |
| 2024 | Wang.Z | Multi-branching temporal convolutional network with tensor data completion for diabetic retinopathy prediction (120) | CNN | Cerner Health Facts (secondary care) | 12.5k cases, 401k controls | ? | Blood tests, 5 comorbidity variables, sex, age, race diabetes duration | Diabetic retinopathy | 70/10/20 | Other imputation variations | N |
| 2024 | Wang.W | Mdpg: a novel multi-disease diagnosis prediction method based on patient knowledge graphs (107) | Graph convolutional network + self-attention | MIMIC-III, MedClin Clinical Diagnosis and Treatment (secondary care) | 7499, 17052 | 10/11 years | ICD-9/10 diagnoses (CCS), medications, predictions, sex, age | Multi | 75/10/15 | GRAM (70), KAME (134), CAMP (150), GNDP (151), Dipole (133), RNN, Graph convolutional network | Y |
| 2024 | Luo.J | Medical Heterogeneous Graph Transformer for Disease Diagnosis (83) | Graph transformer | MIMIC-IV (secondary care) | 9860 | 40,50,60 or 70 codes | Medications, procedures, sex | Myocardial infarction, pneumonia, heart failure, coronary atherosclerosis, cirrhosis, hypertension | 50/20/30 | GCN(152), GAT(153), Transformer(60), HAN(154), Simple-HGN(155), HINormer(156) | N |
| 2024 | Wang.Z | DKGC-LSTM:Fusion of Domain Knowledge to Guide CNN and LSTM for Heart Failure Risk Prediction (85) | CNN + LSTM | MIMIC-IV (secondary care) | 1782 | ? | Laboratory tests, age, height, weight, sex | Heart failure | 80/10/10 | RNN, GRU, LSTM, LSTM-Time, KIT-LSTM(157) | N |

Abbreviations: **ADNI** – Alzheimer’s Disease Neuroimaging Initiative; **ATC** – Anatomical Therapeutic Chemical Classification; **BMI** – Body Mass Index; **CPT** – Current Procedural Terminology; **COPD** – Chronic Obstructive Pulmonary Disorder; **CS** – Cosine similarity; **CPRD** – Clinical Practice Research Datalink; **DAG** – Directed Acyclic Graphs; **DNPR** – Danish National Patient Register; **ECG** – Electrocardiogram; **ED** - Euclidian distance; **FCNN** – Fully Connected Neural Network; **FFN** – Feed-forward Network; **GBT** – Group-based-trajectory Modelling; **GMML** – Geometric mean metric learning; **GPI** – General Product Identifier; **GRU** – Gated Recurrent Unit; **HES** – Hospital Episode Statistics; **ICD** – International Classification of Diseases; **ITML** – Information-theoretic metric learning; **KNN** – K-nearest Neighbours; **LMNNC** – Large margin nearest neighbour classification; **LR** – Logistic Regression; **LSTM** – Long Short-Term Memory; **MIMIC** – Medical Information Mart for Intensive Care; **MLP** – Multi-layer-perceptron; **PD** – Parkinson’s Disease; **PPAD** – Predicting Progression of Alzheimer’s Disease; **RA** – Relative Association; **RF** – Random Forest; **RNN** – Recurrent Neural Network; **RR** – Relative Risk; **SNOMED CT** – Systemized Nomenclature of Medical Clinical Terms; **SCML** – Sparse compositional metric learning; **SVM** – Support Vector Machine.

Footnote: The main list of references can be found accompanying the main text, however additional references found only in this table are stated below, continuing the numeric order as ended in the main script.

1. Lin H, Rong R, Gao X, et al. Disease correlation network: a computational package for identifying temporal correlations between disease states from Large-Scale longitudinal medical records. *JAMIA Open*. 2019 Oct 1;2(3):353. Available from: <https://pmc.ncbi.nlm.nih.gov/articles/PMC6952009/> (Accessed 2025 Jan 31).
2. Nguyen P, Tran T, Wickramasinghe N, et al. Deepr: A Convolutional Net for Medical Records. *IEEE J Biomed Health Inform*. 2016 Dec 01;21(1):22-30. Available from: <https://doi.org/10.1109/JBHI.2016.2633963> (Accessed 2024 Oct 31).
3. Bai T, Egleston BL, Zhang S, et al. Interpretable representation learning for healthcare via capturing disease progression through time. Proceedings of the ACM SIGKDD International Conference on Knowledge Discovery and Data Mining. 2018 Jul 19;43–51. Available from: <https://profiles.foxchase.org/en/publications/interpretable-representation-learning-for-healthcare-via-capturin> (Accessed 2025 Jan 17).
4. Ma F, Chitta R, Zhou J, et al. Dipole: Diagnosis prediction in healthcare via attention-based bidirectional recurrent neural networks. Proceedings of the ACM SIGKDD International Conference on Knowledge Discovery and Data Mining. 2017 Aug 13;Part F129685:1903–11. Available from: <https://dl.acm.org/doi/10.1145/3097983.3098088> (Accessed 2025 Jan 17).
5. Ma F, Chitta R, You Q, et al. KAME: Knowledge-based attention model for diagnosis prediction in healthcare. International Conference on Information and Knowledge Management, Proceedings. 2018 Oct 17;743–52. Available from: <https://dl.acm.org/doi/10.1145/3269206.3271701> (Accessed 2025 Jan 31).
6. Baytas IM, Xiao C, Zhang X, et al. Patient subtyping via time-aware LSTM networks. Proceedings of the ACM SIGKDD International Conference on Knowledge Discovery and Data Mining. 2017 Aug 13;Part F129685:65–74. Available from: <https://dl.acm.org/doi/10.1145/3097983.309799> (Accessed 2025 Jan 20).
7. Kwon BC, Choi MJ, Kim JT, et al. RetainVis: Visual Analytics with Interpretable and Interactive Recurrent Neural Networks on Electronic Medical Records*. IEEE Trans Vis Comput Graph.* 2019 Jan 1;25(1):299–309. Available from: <https://doi.org/10.1109/TVCG.2018.2865027> (Accessed 2025 Jan 20)
8. Gao J, Xiao C, Wang Y, et al. StageNet: Stage-Aware Neural Networks for Health Risk Prediction. The Web Conference 2020 - Proceedings of the World Wide Web Conference, WWW 2020. 2020 Apr 20;530–40. Available from: <https://dl.acm.org/doi/10.1145/3366423.3380136> (Accessed 2025 Jan 20).
9. Choi E, Xiao C, Sun J, et al. MiME: Multilevel Medical Embedding of Electronic Health Records for Predictive Healthcare. *Adv Neural Inf Process Syst*. 2018 Oct 22;2018-December:4547–57. Available from: <https://arxiv.org/abs/1810.09593v1> (Accessed 2025 Jan 20).
10. Kim YJ, Lee YG, Kim JW, et al. Highrisk Prediction from Electronic Medical Records via Deep Attention Networks. *IEEE/ACM Trans Comput Biol Bioinform*. 2021 May-Jun;18(3):1093-1105. Available from: 10.1109/TCBB.2019.2935059. Available from: <https://doi.org/10.1109/TCBB.2019.2935059> (Accessed 2025 Jan 20).
11. Ma L, Gao J, Wang Y, et al. AdaCare: Explainable Clinical Health Status Representation Learning via Scale-Adaptive Feature Extraction and Recalibration. Proceedings of the AAAI Conference on Artificial Intelligence. 2020 Apr 3;34(01):825–32. Available from: <https://ojs.aaai.org/index.php/AAAI/article/view/5427> (Accessed 2025 Jan 17).
12. Pang C, Jiang X, Kalluri KS, et al. CEHR-BERT: Incorporating temporal information from structured EHR data to improve prediction tasks. *Proc Mach Learn Res*. 2021 Nov 10;158:239–51. Available from: <https://arxiv.org/abs/2111.08585v1> (Accessed 2025 Jan 17).
13. Liu S, Li T, Ding H, et al. A hybrid method of recurrent neural network and graph neural network for next-period prescription prediction. *Int J Mach Learn Cybern*. 2020 Dec 1;11(12):2849–56. Available from: <https://pubmed.ncbi.nlm.nih.gov/33727983/> (Accessed 2025 Jan 9).
14. Jin B, Yang H, Sun L, et al. A Treatment Engine by Predicting Next-Period Prescriptions. [KDD '18: Proceedings of the 24th ACM SIGKDD International Conference on Knowledge Discovery & Data Mining](https://dl.acm.org/doi/proceedings/10.1145/3219819). Available from: <https://doi.org/10.1145/3219819.3220095> (Accessed 2025 Jan 20).
15. Ma L, Zhang C, Wang Y, et al. ConCare: Personalized clinical feature embedding via capturing the healthcare context. AAAI Press / International Joint Conferences on Artificial Intelligence; 2020. p. 833–40. Available from: <https://pureportal.coventry.ac.uk/en/publications/concare-personalized-clinical-feature-embedding-via-capturing-the> (Accessed 2025 Jan 20).
16. Luo J, Ye M, Xiao C, et al. HiTANet: Hierarchical Time-Aware Attention Networks for Risk Prediction on Electronic Health Records. Proceedings of the ACM SIGKDD International Conference on Knowledge Discovery and Data Mining. 2020 Aug 23;647–56. Available from: <https://dl.acm.org/doi/10.1145/3394486.3403107> (Accessed 2025 Jan 20).
17. Wang Z, Yan W, Oates T. Time series classification from scratch with deep neural networks: A strong baseline. Proceedings of the International Joint Conference on Neural Networks. 2017 Jun 30;2017-May:1578–85.
18. Mao C, Yao L, Luo Y. MedGCN: Medication recommendation and lab test imputation via graph convolutional networks. *J Biomed Inform*. 2022 Mar 1;127:104000. Available from: <https://doi.org/10.1016/j.jbi.2022.104000>
19. Lu C, Reddy CK, Chakraborty P, et al. Collaborative Graph Learning with Auxiliary Text for Temporal Event Prediction in Healthcare. IJCAI International Joint Conference on Artificial Intelligence. 2021 May 16;3529–35. Available from: <https://arxiv.org/pdf/2105.07542> (Accessed 2025 Sep 3).
20. Chollet F. Xception: Deep Learning with Depthwise Separable Convolutions. Computer Vision and Pattern Recognition. 2016 Nov 6;2017-January:1800–7.
21. Gao J, Wang X, Wang Y, et al. CAMP: Co-Attention Memory Networks for Diagnosis Prediction in Healthcare. Proceedings - IEEE International Conference on Data Mining, ICDM. 2019 Nov 1;2019-November:1036–41. Available from: <https://pureportal.coventry.ac.uk/en/publications/camp-co-attention-memory-networks-for-diagnosis-prediction-in-hea> (Accessed 2025 Jan 31).
22. Li Y, Qian B, Zhang X, et al. Graph Neural Network-Based Diagnosis Prediction. *Big Data.* 2020 Oct 1;8(5):379–90. Available from: <https://pubmed.ncbi.nlm.nih.gov/32783631/> (Accessed 2025 Jan 31).
23. Kipf TN, Welling M. Semi-Supervised Classification with Graph Convolutional Networks. 5th International Conference on Learning Representations, ICLR 2017 - Conference Track Proceedings. 2016 Sep 9. Available from: <https://arxiv.org/pdf/1609.02907> (Accessed 2025 Sep 3)
24. Veličković P, Casanova A, Liò P, et al. Graph Attention Networks. 6th International Conference on Learning Representations, ICLR 2018 - Conference Track Proceedings. 2017 Oct 30. Available from: <https://arxiv.org/pdf/1710.10903> (Accessed 2025 Sep 3).
25. Wang X, Ji H, Cui P, et al. Heterogeneous Graph Attention Network. The Web Conference 2019 - Proceedings of the World Wide Web Conference, WWW 2019. 2019 Mar 18;2022–32. Available from: <https://arxiv.org/pdf/1903.07293> (Accessed 2025 Sep 3).
26. Lv Q, Ding M, Liu Q, et al. Are we really making much progress? Revisiting, benchmarking, and refining heterogeneous graph neural networks. Proceedings of the ACM SIGKDD International Conference on Knowledge Discovery and Data Mining. 2021 Dec 30;21:1150–60. Available from: <https://arxiv.org/pdf/2112.14936> (Accessed 2025 Sep 3).
27. Mao Q, Liu Z, Liu C, et al. HINormer: Representation Learning On Heterogeneous Information Networks with Graph Transformer. ACM Web Conference 2023 - Proceedings of the World Wide Web Conference, WWW 2023. 2023 Apr 30;599–610. Available from: <https://dl.acm.org/doi/pdf/10.1145/3543507.3583493> (Accessed 2025 Sep 3).
28. Liu LJ, Ortiz-Soriano V, Neyra JA, et al. KIT-LSTM: Knowledge-guided Time-aware LSTM for Continuous Clinical Risk Prediction. Proceedings - 2022 IEEE International Conference on Bioinformatics and Biomedicine, BIBM 2022. 2022;2022:1086–91. Available from: <https://pubmed.ncbi.nlm.nih.gov/37131483/> (Accessed 2025 Sep 3).

**Supplementary table 5:** Definitions of performance metrics used by studies in this review.

| **Performance metric** | **Definition** |
| --- | --- |
| Area under the curve (AUC) / Area under the receiver operating characteristic curve (AUROC) | The ROC curve plots sensitivity against 1-sensitivity (false positives) where 0.5 represents a random guess, and 1 represents perfect classification. |
| Precision (positive predictive value) | True positives over all predicted positive values. |
| Recall (aka sensitivity)  Recall@k | True positives over all of the values that should have been positive.  The proportion of relevant predictions in the top k highest-ranked predictions or recommendations. |
| F1 score | The harmonic mean of the precision and recall scores: (2*precision*recall) / precision + recall. |
| Area under the precision-recall curve (AU-PR) | Plots precision against recall, on one extreme the model predicts more positives, leading to high recall but low precision. |
| Accuracy | True positives + true negatives / (true positives + true negatives + false positives + false negatives). |
| RMSE (root mean square error) | The standard deviation of the residuals i.e. how far predictions fall from true values. |
